# Endocrine-Disrupting Chemical Exposures in Pregnancy: a Sensitive Window for Later-Life Cardiometabolic Health in Women

**DOI:** 10.1007/s40471-021-00272-7

**Published:** 2021-08-09

**Authors:** Emily S. Barrett, Susan W. Groth, Emma V. Preston, Carolyn Kinkade, Tamarra James-Todd

**Affiliations:** 1 Environmental and Occupational Health Sciences Institute, Rutgers University, Piscataway, NJ 08854, USA; 2 Department of Biostatistics and Epidemiology, Rutgers School of Public Health, Piscataway, NJ 08854, USA; 3 University of Rochester School of Nursing, Rochester, NY 14642, USA; 4 Department of Environmental Health, Harvard T. H. Chan School of Public Health, 665 Huntington Ave., Bldg. 1, 14th Floor, Boston, MA 02120, USA; 5 Department of Epidemiology, Harvard T. H. Chan School of Public Health, Boston, MA 02115, USA

**Keywords:** Pregnancy, Cardiovascular disease, Hypertensive disorders of pregnancy, Obesity, Endocrine-disrupting chemicals

## Abstract

**Purpose of Review:**

Pregnancy can be seen as a “stress test” with complications predicting later-life cardiovascular disease risk. Here, we review the growing epidemiological literature evaluating environmental endocrine-disrupting chemical (EDC) exposure in pregnancy in relation to two important cardiovascular disease risk factors, hypertensive disorders of pregnancy and maternal obesity.

**Recent Findings:**

Overall, evidence of EDC-maternal cardiometabolic associations was mixed. The most consistent associations were observed for phenols and maternal obesity, as well as for perfluoroalkyl substances (PFASs) with hypertensive disorders. Research on polybrominated flame retardants and maternal cardiometabolic outcomes is limited, but suggestive.

**Summary:**

Although numerous studies evaluated pregnancy outcomes, few evaluated the postpartum period or assessed chemical mixtures. Overall, there is a need to better understand whether pregnancy exposure to these chemicals could contribute to adverse cardiometabolic health outcomes in women, particularly given that cardiovascular disease is the leading cause of death in women.

## Introduction

During pregnancy, cardiometabolic, vascular, endocrine, and immune adaptations radically transform maternal physiology to support and nurture the developing fetus [1–3]. Over the course of a typical pregnancy, weight accumulation, lipid levels, inflammation, and insulin resistance increase, accompanied by decreases in vascular resistance [1–4]. A greater appreciation of the magnitude of these physiological changes has led to the conceptualization of pregnancy as a “stress test,” with the mother’s ability to appropriately recalibrate to (and later, recuperate from) these changing demands providing a window into underlying cardiometabolic health [5–8]. From this perspective, pregnancy complications, such as hypertensive disorders of pregnancy (HDP), may be indicators of underlying metabolic and cardiovascular dysfunction, as well as predictors of future cardiometabolic disease risk. Additionally, overweight and obesity prior to pregnancy and high gestational weight gain (GWG) contribute to pregnancy complications [9], with implications for future maternal health ramifications, such as weight retention [10, 11], type 2 diabetes (T2D), hypertension [10, 12], metabolic syndrome, and negative metabolic profiles following pregnancy [13]. All of these outcomes are, in turn, associated with future cardiovascular disease (CVD), the leading cause of death in women [14, 15].

**Fig. 1:**
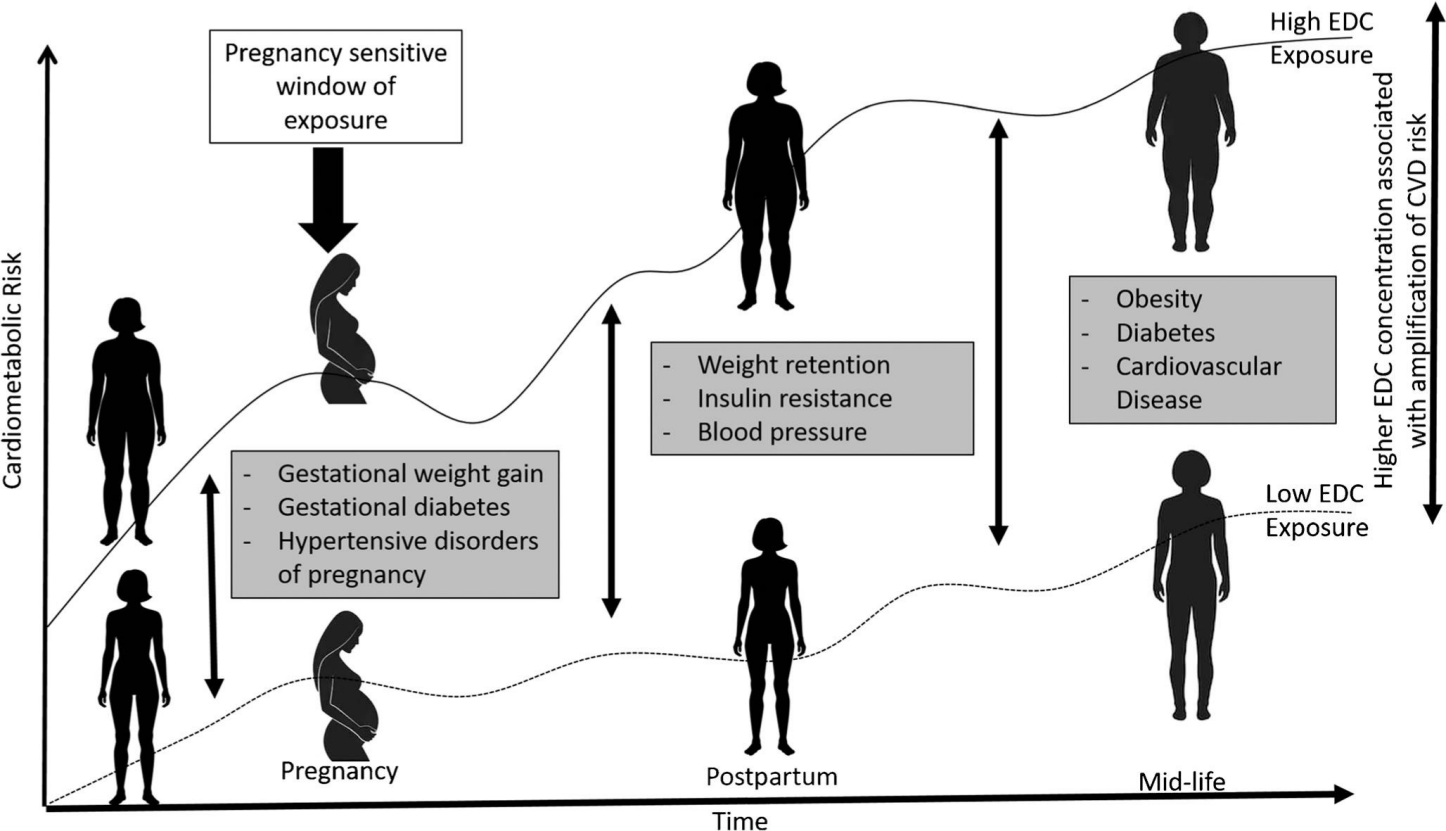
Conceptual model for pregnancy as a sensitive window for EDC exposure as it relates to later-life maternal cardiometabolic health

More recently, a body of work has emerged suggesting that endocrine-disrupting chemicals (EDCs) may dysregulate physiological adaptations during pregnancy, resulting in an increased risk of complications and adverse outcomes for the mother and child. This premise is supported by a large body of work in animal models and non-pregnant adults indicating that EDCs interfere with metabolic activity through effects on adipocyte number and size, metabolic set points, and hormonal pathways [16, 17] (Fig. 1). Given the growing evidence that these chemicals may have long-lasting metabolic effects, an important next step is evaluating their impact on maternal metabolic health during pregnancy and beyond. The objective of this review is to summarize the growing body of epidemiological evidence evaluating EDC exposure in pregnancy in relation to maternal cardiometabolic health outcomes during and after pregnancy. We focus on two important cardiometabolic outcomes that have been assessed in this context across multiple studies: HDP and maternal obesity during and after pregnancy. Both outcomes are associated with later-life CVD risk in women [18, 19]. To date, four classes of synthetic EDCs have been studied in relation to these outcomes and are considered in this review: phthalates, phenols, perfluoroalkyl substances (PFASs), and polybrominated diethyl ethers (PBDEs). This burgeoning area of research highlights the need to consider pregnancy as a sensitive window of environmental chemical exposure as it relates to women’s long-term health.

## Methods

### Search Strategy

We conducted a scoping review using electronic literature searches in the PubMed database for original research articles with publication dates through May 27, 2020. We performed two separate searches, combining our set of exposure terms with each set of outcome category terms (i.e., hypertensive disorders of pregnancy, and maternal weight/adiposity-related outcomes). We used a combination of Medical Subject Headings (MeSH) terms and free text title or abstract terms (tiab). Specific search terms and strategies are included in Supplemental Table 1. In addition to our PubMed search, we manually crosschecked listed references within identified articles for additional relevant studies not captured in our database search.

### Study Screening and Selection

We considered original epidemiologic studies in pregnant or postpartum women relating exposure to one or more EDCs of interest (i.e., phenols, phthalates, PFASs, flame retardants [PBDEs], and organophosphate flame retardants [OPFRs]) to one or both of our outcome categories (i.e., HDP and maternal obesity). Although OPFRs were included in the initial search, no relevant studies were identified; thus, they were not considered further. After removing duplicate articles, we performed an initial screening of the articles from our database searches by reviewing article titles and abstracts. We obtained full-texts for all remaining articles (excluding duplicates), which were then screened for relevance. We excluded articles that were conducted on animals, focused on populations that were not pregnant or postpartum, had irrelevant outcomes or exposures, or were irrelevant study types (e.g., case reports, review articles, editorials). Papers that were not available in English or in full-text were excluded as well.

### Summary of Search Results

We identified 989 articles in our initial PubMed search. After removing duplicates and screening titles and abstracts for relevance, we identified 29 potential articles for which the full-texts were reviewed for relevance. Ultimately 18 articles on HDP, and 9 articles on maternal weight and obesity-related outcomes were included in the review.

## Results: EDCs and Maternal Health During Pregnancy and the Postpartum

### Hypertensive Disorders of Pregnancy

HDP, including gestational hypertension, chronic hypertension, pre-eclampsia, eclampsia, and HELLP syndrome, are a group of high blood pressure disorders that pose serious risks to both mother and fetus. Women who experience these disorders during pregnancy are at increased risk of poor downstream cardiometabolic health including T2D, hypertension, hypercholesterolemia, and CVD [7, 20–23]. HDP incidence is rising [24–26], demonstrating the importance of identifying potentially modifiable risk factors. In addition to lifestyle factors, it has been suggested that environmental chemicals may play an important role. Indeed, some research in non-pregnant populations has suggested that the EDCs of interest may be associated with blood pressure and hypertension [27–29]. Here we review the growing epidemiological literature on these exposures in relation to maternal cardiometabolic health in pregnancy and the postpartum (Table 1).

#### Phthalates

Four published studies have examined associations between phthalates and HDP with inconsistent results. Two studies reported a positive association between phthalate metabolites and HDP. In the US Health Outcomes and Measures of the Environment (HOME) study (*n*=369), mid-gestation mono-benzyl phthalate (MBzP) was associated with higher diastolic blood pressure (DBP) in mid- and late gestation as well as increased risk of developing pregnancy-induced hypertensive diseases (RR=2.92, 95% CI 1.15–7.41) [48]. A case-control study nested within the Boston LIFECODES study (50 pre-eclampsia cases, 431 controls) observed that higher early pregnancy mono-ethyl phthalate (MEP) was associated with subsequent development of pre-eclampsia (hazard ratio=1.72; 95% CI:1.28, 2.30). Higher di(2-ethylhexyl) phthalate (DEHP) metabolite levels across pregnancy were additionally associated with increased risk of pre-eclampsia, with stronger associations in women carrying female fetuses [33]. By contrast, in a small European study (*n*=152), higher MEP and monoisobutyl phthalate (MiBP) were associated with lower blood pressure in pregnancy [32]. In the largest study on this topic, Generation R (*n*=1396), no consistent associations between early pregnancy urinary phthalate metabolite concentrations and blood pressure, gestational hypertension, or pre-eclampsia were observed [31]. However, even this study may have been underpowered to examine pre-eclampsia (*n*=24 cases).

#### Phenols

The literature on phenols in relation to blood pressure and HDP is similarly mixed. In general, beyond bisphenol A (BPA), few studies have evaluated phenols and HDP. In two large pregnancy cohorts, Generation R (*n*=1233) and Maternal-Infant Research on Environmental Chemicals (MIREC) (*n*=1909), overall, early pregnancy BPA was not associated with blood pressure or HDP, though effect modification by parity was reported in the latter [30, 31]. Effect modification of the relationship between phenols and hypertensive outcomes by fetal sex has also been reported, though the sex-specific effects have not been consistent across studies [33, 35].

Two additional small case-control studies (*n*=173 and *n*=58) have examined BPA in blood and placental tissue in women with and without pre-eclampsia, reporting higher BPA levels in cases [34, 37]; however, blood and placental tissue are non-preferred matrices for BPA assessment due to non-persistence of BPA and potential for contamination [36], and reverse causation is also possible. Finally, Warembourg et al. (*n*=152) reported that higher urinary concentrations of ethylparaben, methylparaben, BPA, and triclosan (TCS) were associated with lower maternal systolic and diastolic blood pressure in the second and third trimesters [32]. For example, a doubling of BPA concentrations was associated with a 0.91 mmHg decrease in systolic blood pressure (SBP) (95% CI: −1.65, −0.17).

#### PFAS

The earliest work on PFASs and HDP came from the US C8 cohort, who were exposed to elevated perfluorooctanoic acid (PFOA) through drinking water contamination [49]. In a nested case-control study using birth record data from 224 participants with pregnancy-induced hypertension and a sample of term birth controls, no differences in modeled estimates of serum PFOA were observed [49]. In a larger analysis from the same cohort based on modeled PFOA concentrations and self-reported pregnancy history (*n*=11,737 pregnancies), an interquartile increase in log-transformed PFOA was associated with slightly increased odds of pre-eclampsia (OR: 1.13; 95% CI: 1.00–1.28); however, the lack of direct exposure data and self-reported outcome data were notable limitations [43]. An additional analysis in the subset of women with measured (rather than estimated) serum PFAS examined pregnancy outcomes in the 5 years prior to study participation and reported weak associations between PFOA concentrations and pre-eclampsia (aOR: 1.3, 95% CI: 0.9, 1.9) as well as PFOS concentrations (aOR: 1.3, 95% CI: 1.1, 1.7) [44].

Several international cohorts have examined these associations as well. In a cross-sectional Chinese study (*n*=687), cord blood concentrations of perfluorobutane sulfonate (PFBS), perfluorohexanesulfonate (PFHxS), and perfluoroundecanoic acid (PFUA) were all associated with pre-eclampsia in elastic net penalty regression models; however, in fully adjusted models, only PFBS concentrations were associated with odds of pre-eclampsia (aOR: 1.81, 95% CI: 1.03, 3.17) and higher HDP (aOR: 1.64, 95% CI: 1.09, 2.47) [45]. By contrast, a follow-up prospective cohort study by the same research team (*n*=3220) did not observe any consistent associations between early pregnancy PFAS concentrations and gestational hypertension, pre-eclampsia, or overall HDP [40].

In a study of nulliparous participants in the Norwegian Mother and Child (MoBa) cohort (466 pre-eclampsia cases, 510 non-cases), although there was an unexpected inverse association between pre-eclampsia and PFUA concentrations, overall results did not support a relationship between PFASs and hypertensive disorders [38]. By contrast, in the Swedish SELMA study (*n*=1773 including 64 women with pre-eclampsia), a doubling of PFOS and perfluorononanoic acid (PFNA) concentrations in early pregnancy was associated with 38% (95% CI: 1.01, 1.89) and 53% (95% CI: 1.07, 2.20) increased risk of pre-eclampsia, respectively [42]. Finally, the Canadian MIREC study (*n*=1739) observed fetal sex-specific associations between PFAS concentrations and HDP [41]. A doubling of PFHxS levels was associated with higher odds of pre-eclampsia in women carrying female fetuses, whereas among women carrying male fetuses, perfluorooctanesulfonate (PFOS) and PFHxS were associated with increased odds of gestational hypertension. Overall, higher PFAS concentrations were associated with increases in diastolic blood pressure.

#### PBDEs

To our knowledge, only two studies have examined PBDEs in relation to HDP. In the Longitudinal Investigation of Fertility and the Environment (LIFE) study, pre-conception concentrations of PBDEs were measured; given the persistence and long half-life of many PBDEs in the body, pre-conception levels are likely to be a reasonable approximation of concentrations in pregnancy [39, 47]. In that study, a 1 SD increased in ln-transformed serum BDE-66 was associated with a 56% increased odds of gestational hypertension (95% CI: 0.93, 2.64); however, >80% of values were below the limit of detection and only 27 of 231 women had gestational hypertension, suggesting limited power to detect associations [46]. In a smaller Iranian study (45 pre-eclampsia cases, 70 controls), four of eight PBDE congeners measured (28, 47, 99, 153) were significantly higher among cases in unadjusted models. After adjustment for covariates, the odds of pre-eclampsia were significantly associated with total PBDE concentrations (aOR: 2.19; 95% CI: 1.39, 3.45) [50].

### Maternal Weight/Obesity-Related Outcomes

Compared to HDP, maternal obesity and excess GWG have attracted relatively little attention in the context of EDCs. Overweight and excess GWG increase risks for both adverse birth outcomes, as well as weight retention in the postpartum [11], a vicious cycle which contributes to increasing baseline weight over successive pregnancies and, ultimately, elevated cardiometabolic risk [51]. Excess GWG refers to weight gain above the recommended amount for a woman’s pre-pregnancy BMI [52]. Evidence from animal models and epidemiological studies of non-pregnant adults suggests a potential role of EDCs in weight gain, adipogenesis, and metabolic dysregulation [16, 17]. It is plausible, therefore, that pregnancy, a period characterized by extensive physiological changes that promote weight gain and fat deposition [51], may be a period of particular vulnerability to EDC exposure. Numerous studies have considered maternal body mass index (BMI) and GWG as predictors of EDC concentrations [53]; however, few have considered the reverse scenario that EDCs contribute to maternal BMI, GWG, and weight retention. Here we briefly describe current research on these outcomes in relation to the EDCs of interest (Table 2).

#### Phthalates

Four studies have examined maternal phthalate exposure in relation to BMI, GWG, and/or postpartum weight retention. Bellavia et al. evaluated cross-sectional associations between first trimester phthalate metabolite concentrations (measured median 9.9 weeks) and first trimester BMI (assessed at the same time period) (*n*=347 women). This study also prospectively assessed associations between phthalates measured at that same time point and GWG gain assessed from the first trimester to early second trimester (median difference between the two pregnancy weight measurements was 7.4 weeks) [55]. Their results indicated that increased concentrations of several metabolites (namely MEP, MBzP, mono(3-carboxypropyl) phthalate [MCPP], and ΣDEHP) were associated with a higher BMI in the first trimester suggesting that women with higher concentrations had higher pre-pregnancy BMIs. Higher MEP concentrations were associated with higher GWG from the first prenatal visit until early 2nd trimester, while inverse associations were observed for ΣDEHP. More recently, the Generation R study (*n*=1213) observed no associations between phthalate metabolite concentrations in early pregnancy (median 13.1 weeks) or mid-pregnancy (median 20.4 weeks gestation) at total and total GWG [54]. Limited inverse associations were observed between the di-n-octylphthalate (DNOP) metabolites and low molecular weight phthalate metabolites and weight gain during mid-to-late pregnancy.

In one of the few studies to examine EDCs in relation to postpartum weight retention, Rodriguez-Carmona et al. followed 178 women from the Mexican pregnancy cohort, Early Life Exposure in Mexico to Environmental Toxicants (ELEMENT), and assessed maternal body weight 5 times in the first year postpartum and at follow-up visits at approximately 7 and 10 years postpartum [57]. Overall, a one-unit increase in log-transformed MCPP concentrations in pregnancy was associated with 0.33 kg (95% CI: 0.09, 0.56) increase in weight gain per postpartum year, whereas a one-unit increase in log-transformed MBzP in pregnancy was associated with 0.21 kg (95% CI: −0.38, −0.03) decrease in weight gain - per postpartum year. However, data collection on additional pregnancies (including associated weight gain) during the follow-up period was limited and presents a potentially important source of error. A second analysis of data from the same study population (*n*=199) assessed effects over a shorter time frame, namely the first year postpartum [61]. They observed that women with a higher exposure to EDCs during pregnancy lost less weight during the first postpartum year. For example, an interquartile increase in ΣDEHP was associated with 1.01 kg (95% CI: 0.41, 1.61) less weight loss at 1 year postpartum [61].

#### Phenols

To date, four studies have examined phenols in relation to maternal perinatal weight gain and loss. In the Generation R study (*n*=1213), high total bisphenol (combined molar concentrations of BPA, BPS, BPF) and BPS concentrations in early pregnancy were associated with less total GWG. In stratified analyses, associations were only observed in normal weight women; however, there were insufficient numbers in other BMI categories to assess associations [54]. The MIREC study (*n* = 1795) reported on GWG in relation to triclosan, but no other phenols [56]. Although mean first trimester triclosan was highest in women who went on to meet or exceed Institute of Medicine (IOM) recommendations for GWG [58], in adjusted models, triclosan concentrations were not associated with total GWG. Wen et al. examined paraben exposure and GWG in each trimester (*n*=613), observing that concentrations of methyl (MeP), ethyl (EtP), and propyl (PrP) paraben as well as the Σparabens were positively associated with trimester-specific GWG. All paraben concentrations (individual and sum) in the first trimester were associated with first trimester GWG rates; these early pregnancy associations were stronger than those observed in the second or third trimesters [59]. Effect modification by maternal pre-pregnancy BMI was again noted, such that associations were stronger in overweight/obese women compared to normal/underweight women. Finally, to our knowledge, only one study (ELEMENT, *n*=199) has examined phenols in relation to postpartum weight retention, observing that BPA concentrations during pregnancy were associated with less weight loss over the first postpartum year [61].

#### PFAS

Three studies have examined PFAS in relation to GWG. In the Avon Longitudinal Study of Parents and Children (ALSPAC, *n* = 905), Marks et al. observed no associations between serum PFAS (median 18 weeks gestation) and absolute weight gain (measured prior to 18 weeks gestation and after 28 weeks gestation) [60]. However, in stratified analyses, higher PFNA concentrations were associated with higher GWG in under/normal weight women. Under/normal weight women (BMI < 25 kg/m^2^) also appeared to be vulnerable to PFAS exposure in the LIFE study (*n*=218). In the under/normal weight group, pre-conception plasma PFOS concentrations were positively associated with the area under the curve of GWG, calculated as the sum of trapezoids resulting from consecutive measures of weight gain (relative to baseline weight) across pregnancy (*ß*=280.29, 95% CI: 13.71, 546.86). However, no associations were observed for women with a BMI = 25 kg/m^2^ nor for the other six PFASs studied [62]. In addition, PFAS concentrations were not associated with total GWG, nor odds of inadequate or excessive weight gain. Somewhat consistent with these results were findings from the MIREC cohort (*n*=1723) suggesting that higher maternal first trimester PFOS concentrations were associated with greater GWG per week across the second and third trimesters [63]. Again, no associations were noted for the other PFASs studied.

#### PBDEs

Only one study to our knowledge has evaluated PBDEs and maternal weight outcomes. In the LIFE study (*n*=218), in addition to PFAS, Jaacks et al. assessed pre-conception plasma PBDE concentrations and GWG in pregnancy [62]. Of 10 PBDEs measured, most notably, higher BDE-99 concentrations were associated with greater total GWG (*ß*=1.07, 95% CI: 0.00, 2.14). In addition, BDE-66 concentrations were negatively associated with the area under the curve of GWG among women who were overweight or obese (*ß*=−376.04, 95% CI: −734.05, −19.03). Jaacks et al. interpret the AUC as “additional pound-days carried by a women during her pregnancy relative to remaining at her pre-pregnancy weight,” suggesting that women with higher BDE-66 concentrations had significantly fewer “pound-days” compared to women with lower BDE-66 concentrations. Of note, 87% of samples had BDE-66 concentrations below the LOD. No additional associations were noted.

## Conclusions/Future Directions

In this scoping review, we evaluated four classes of EDCs to assess their association with maternal cardiometabolic outcomes as they relate to exposure during pregnancy. This continues to be an emerging area of research, with more studies in the area of maternal hypertensive disorders of pregnancy compared to maternal obesity. Few studies evaluated related postpartum outcomes, a major gap in the literature given the rapid metabolic change that occurs during this period. To date, the primary chemical class that has been evaluated is phthalates. Although results have been mixed, some studies have observed positive associations with certain phthalate metabolites (i.e., MBzP and MEP) and there is a clear need for larger studies with greater power. In addition, the majority of studies have found associations between phthalates with weight measures, with MBzP consistently implicated. A number of studies have evaluated PFAS and maternal cardiometabolic outcomes, with PFHxS identified as a chemical of concern. In fact, PFHxS was associated with risk of maternal dysglycemia as well as HDP. Other PFASs were also highlighted as potential risk factors for HDP, though findings were inconsistent. Mixed results were observed for BPA as well, which most consistently associated with maternal weight changes. Finally, only a few studies evaluated PBDEs; however, results suggest that these chemicals may be important to consider with respect to maternal cardiometabolic health outcomes.

Many of the studies reviewed were sizeable prospective cohort studies. However, quite a few were small case-control studies with limited power. That said, several of the larger prospective cohort studies also had limited power, given the fact that they were low-risk populations for these cardiometabolic outcomes and had relatively few participants with the relevant outcomes (such as pre-eclampsia). Only a few studies evaluated associations in high-risk populations or conducted stratified analyses to evaluate higher risk subsets of the population. Interestingly, these studies found differing associations, suggesting the need to consider other mitigating factors such as family history of diabetes, maternal obesity in the context of gestational diabetes and HDP, and race/ethnicity when evaluating these associations. Of concern, some studies evaluated exposure following diagnosis of the cardiometabolic outcome, putting into question temporality and raising difficulty with interpretation of results. More generally, variation in timing of exposure assessment makes it difficult to draw conclusions given that exposures across different windows of pregnancy may affect maternal physiology and disease risk in substantially varied ways. This is particularly true for nonpersistent chemicals, such as phthalates and phenols, which have half-lives in hours or days. Of note, few studies evaluated prevalent chemicals, such as TCS and OPFRs. Importantly, few studies considered chemical mixtures, an important emerging area of research. Finally, the preponderance of the evidence to date comes from a small number of cohorts, such as MIREC, Generation R, LIFECODES, and the LIFE studies, though additional studies (including in China and Mexico), are now beginning to evaluate these associations as well. Given the demographic composition of the studies above, which include a majority of women from White, North American, or European backgrounds, there is a need for more diverse cohorts to better understand the impact of these chemicals on maternal cardiometabolic health. Environmental health cohorts specifically designed to examine maternal health as a primary outcome (rather than being secondary to child developmental outcomes) are warranted.

Maternal cardiometabolic health (and complications) in pregnancy has been linked to a number of later-life chronic diseases. Interventions are currently underway that involve lifestyle and diet modifications, as well as pharmaceutical interventions, such as daily low-dose aspirin to reduce cardiovascular disease risk after pregnancy. However, these interventions are unable to completely eliminate risk [64], perhaps because they do not address critical underlying factors that contribute to pregnancy complications. Environmental toxicants may be one such factor. Removing or reducing exposure to environmental toxicants—whether before, during, or after pregnancy— may improve maternal cardiometabolic health. Several strategies could be implemented to bolster research in this area:

1. Leverage ongoing pregnancy/birth cohorts with environmental health data to conduct long-term follow-up of mothers for chronic disease outcomes
2. Standardize data collection of maternal health to include cardiometabolic outcomes, such as blood pressure, fasting and postprandial glucose measurements, lipid levels, adverse pregnancy outcomes, and anthropometric measurements
3. Consider the importance of timing of exposure, including the different windows of susceptibility during pregnancy by including measurements during the 1st, 2nd, and 3rd trimesters
4. Consider the extended postpartum period (up to several years after birth), given its significance for a metabolic reset that is critical for return to a normal, non-pregnant metabolic state
5. Consider the social, cultural, and behavioral drivers of environmental exposures in the context of pregnancy and the postpartum period
6. Identify the mechanisms that could be involved in pregnancy and the postpartum as sensitive windows of exposure to determine whether elevated disease risks are temporary or permanent
7. Determine potential strategies for interventions to reduce toxicant exposures that impact pregnancy, postpartum, and later-life cardiometabolic health risk
8. Incorporate environmental assessments as a part of obstetrics, gynecologic, and primary care to assess risk as a strategy for environmental disease prevention

As a discipline, environmental health has made tremendous strides in understanding the links between environmental risk factors and adverse health outcomes. Research in the areas of prenatal exposure and child health outcomes is particularly robust. However, understanding of other life-course sensitive periods is less well-studied, particularly as it relates to women’s health. Many environmental health pregnancy/birth cohorts have key exposure data that would enable evaluating pregnancy as a sensitive period for later-life cardiometabolic health risk in women. However, many of these cohorts have not captured maternal exposure and outcome information beyond pregnancy. Furthermore, as we consider the pregnancy period, it is just as critical to consider the postpartum period—a time of rapid metabolic transition. With CVD being the leading cause of death in women, determining the extent to which pregnancy and postpartum exposure to these prevalent environmental chemicals plays a role in the development of adverse cardiometabolic outcomes during and after pregnancy is imperative. If pregnancy and the postpartum prove to be sensitive windows of exposure, interventions could be developed to reduce exposure to these EDCs during sensitive periods with implications for reducing HDP and maternal obesity—critical factors that contribute to future CVD risk in women.

## Supplementary Material

Supplementary Material

## Figures and Tables

**Table 1:** Pregnancy EDC exposure and maternal hypertensive disorders of pregnancy

Author (year)	Study sample (and location)	Study design	Exposure measures and timing	Outcome measures and timing	Main findings

• Philips (2019) [30]	1396 (Netherlands); *n*=40 with GHTN, *n*=24 with PE	Prospective cohort (Generation R)	Urinary BPA phthalate metabolites at median 13 weeks gestation	SBP and DBP in each trimester; clinical diagnosis of gestational hypertensive disorders	• No consistent associations with BP or gestational hypertensive disorders
Warembourg (2019) [31]	152 (multi-site, Europe)	Prospective cohort (HELIX)	Urinary phenols and phthalate metabolites at 18 and 32 weeks gestation	SBP and DBP across pregnancy	• Some associations between higher exposures and decreased SBP (e.g., *β*=−0.75mmHg per doubling of MEP) • Stronger inverse associations in mid-pregnancy
• Cantonwine (2016) [32]	50 PE cases, 431 controls (Massachusetts, USA)	Nested case-control (LIFECODES)	Urinary phthalate metabolites and BPA at 10 weeks gestation	PE defined as SBP ≥140 mmHg or DBP ≥90 mmHg along with positive urinary protein test	• IQR increase in MEP at GA 10 weeks associated with 1.72 (95% CI: 1.28, 2.30) aHR • IQR increase in BPA at GA 10 weeks associated with 1.53 (95% CI: 1.04, 2.25) aHR • DEHP across gestation associated with elevated hazard of PE (stronger associations in female fetuses)
Werner (2015) [33]	369 (Ohio, USA); *n*=13 with GHTN; *n*=21 with PE; *n*=2 with HELLP syndrome	Prospective cohort (HOME)	Urinary phthalate metabolites at 16 and 26 weeks gestation	Clinical gestational hypertensive diagnoses (gestational hypertension, pre-eclampsia, eclampsia, or HELLP syndrome); SBP and DBP (from medical record at <20 weeks GA)	• Compared to first tertile, women in highest MBzP tertile at 16 weeks GA had DBP 2.2 and 2.8 mmHg higher DBP at <20 weeks and ≥20 weeks gestation, respectively • Compared to first tertile, women in highest MBzP tertile at increased risk of developing PIH (RR: 2.92, 95% CI: 1.15, 7.41) • No association with other phthalate metabolites.
Liu (2019) [34]	644 (China)	Prospective cohort (Wuhan)	Urinary phenols in each trimester	SBP and DBP in each trimester	• Among all women, no associations observed • In women carrying males, some evidence of positive associations between TCS, BP-1, 4-OH-BP, and Σbenzophenones, and SBP (but not DBP) • In women carrying females, only a weak inverse association between TCS and DBP
Camara (2018) [35]	1909 (Canada); *n*=128 with GHTN; *n*=57 with PE	Prospective cohort (MIREC)	Urinary BPA and TCS in trimester 1	SBP and DBP at prenatal visits in each trimester; GHTN: SBP ≥ 140 mmHg and/or DBP ≥ 90 mmHg at ≥ 20 weeks; PE: GHTN plus proteinuria or related maternal complications	• BPA and TCS not associated with odds of hypertension or pre-eclampsia • Some evidence of effect modification by parity
Ye (2017) [36]	74 PE cases, 99 controls (China)	Nested case-control	Serum BPA at 16–20 weeks gestation	PE diagnosis in medical record	• Higher serum BPA concentrations in women with pre-eclampsia compared to controls (aOR: 16.5, 95% CI: 5.4, 49.9)
Leclerc (2014) [37]	23 PE cases, 35 controls (Canada)	Case-control	Serum, umbilical cord, and placental BPA at delivery	PE diagnosis	• Higher BPA in PE placentas compared to controls (*p*=0.04), no differences in serum levels, no adjustment for covariates.
Huo (2020) [38]	3220 (China); *n*=125 with HDP	Prospective cohort (Shanghai Birth Cohort)	Plasma PFAS at median 15 weeks gestation	Medical record diagnoses of GHTN, PE, overall HDP (GHTN or PE)	• No PFAS was associated with GHTN, PE or HDP, no effect modification by parity
Borghese (2020) [39]	1739 (Canada); *n*=127 with GHTN (without PE); *n*=49 with PE	Prospective cohort (MIREC)	Plasma PFAS at mean 11.6 weeks gestation	SBP and DBP at prenatal visits in each trimester; GHTN: SBP ≥ 140 mmHg and/or DBP ≥ 90 mmHg at ≥ 20 weeks; PE: GHTN plus proteinuria or related maternal complications	• 1.32 times (95% CI: 1.03, 1.70) increased odds of PE per doubling of PFHxS concentrations • Higher concentrations of PFOA, PFOS, and PFHxS associated with increased DBP across pregnancy; PFOA and PFHxS were associated with SBP • Some evidence of effect modification by fetal sex
Huang (2019) [40]	687 (China); *n*=42 with HDP	Cross-sectional	Umbilical cord plasma PFAS	Medical record diagnoses of GHTN PE, HDP	• PFBS associated with higher odds of HDP (aOR: 1.64, 95% CI: 1.09, 2.47) and PE (aOR: 1.81, 95% CI: 1.03, 3.17) • PFHxS and PFUA associated with PE in elastic net penalty regression, but not logistic regression models • No associations observed for PFOA or PFOS
• Wikstrom (2019) [41]	1773 (Sweden); *n*=64 with PE	Prospective cohort (SELMA)	Serum PFAS at median 10 weeks gestation	PE diagnosis in medical birth register	• Doubling of PFOS and PFNA associated with 38% and 53% increased risk of pre-eclampsia, respectively • 2.68 times the odds (95% CI: 1.17, 6.12) of PE among highest quartile of PFOS compared to lowest • Stronger associations in nulliparous women • No associations for other PFAS
Starling (2014) [42]	466 PE cases, 510 controls (Norway)	Nested case-control (MoBa)	Plasma PFAS in pregnancy	PE in medical record	• No consistent associations of PFAS concentrations with PE • Inverse association between perfluoroundecanoic acid concentrations and greater risk of PE (HR 0.55, 95% CI: 0.38, 0.81) for highest compared to lowest quartile
Savitz (2012a) [43]	224 PIH cases, 3616 controls (Ohio, USA)	Nested case-control (C8 Health Project)	Serum PFOA estimated from pharmacokinetic and environmental models, residence history	PIH	• No association between PFOA and PIH (aOR 1.02, (95% CI: 0.86, 1.21)
Savitz (2012b) [44]	11737 (Ohio, USA); *n*=730 with PE	Cross-sectional (C8 Health Project)	Serum PFOA estimated from pharmacokinetic and environmental models, residence history	Self-reported PE from 1990 to enrollment (2005–2006)	• IQR increase in PFOA associated with 1.13 (95% CI: 1.00–1.28) adjusted odds of PE
Stein (2009) [45]	5663 (Ohio, USA); *n*=156 with PE	Cross-sectional (C8 Health Project)	Serum PFOA and PFOS at enrollment	Self-reported PE in the previous 5 years	• PE weakly associated with PFOA (aOR: 1.3, 95% CI: 0.9, 1.9) and PFOS (aOR: 1.3, 95% CI: 1.1, 1.7)
Eslami (2016) [46]	45 PE cases, 70 controls (Iran)	Case-control	Trimester 3 serum PBDEs	PE clinical diagnosis	• Total PBDE concentrations were positively associated with odds of PE (OR: 2.19, 95% CI: 1.39, 3.45), though associations attenuated after adjustment for PCBs.
Smarr (2016) [47]	258 (Michigan and Texas, USA); *n*=27 with GHTN	Prospective cohort (LIFE)	Pre-conception serum PBDEs	Self-reported GHTN at ≥ 24 weeks gestation	• Serum PBDE concentrations were not associated with odds of GHTN • Higher BDE-66 suggestively associated with greater odds of GHTN (OR 1.56; 95% CI: 0.93, 2.64)

*ACOG*, American College of Obstetrics and Gynecology; *aOR*, adjusted odds ratio; *BDE-66*, 2,3′,4,4′-tetrabromodiphenyl ether; *BP*, blood pressure; *BPA*, bisphenol A; *DBP*, diastolic blood pressure; *DEHP*, di(2-ethylhexyl) phthalate; *GA*, gestational age; *GHTN*, gestational hypertension; *HDP*, hypertensive disorders of pregnancy; *HELLP*, hemolysis, elevated liver enzymes, low platelet count; *HR*, hazard ratio; *IQR*, interquartile range; *MBzP*, mono-benzyl phthalate; *MECPP*, mono(2-ethyl-5-carboxypentyl) phthalate; *MEHHP*, mono(2-ethyl-5-hydroxyhexyl) phthalate; *MEHP*, mono(2-ethylhexyl) phthalate; *MEOHP*, mono-(2-ethyl-5-oxohexyl) phthalate; *MEP*, mono-ethyl phthalate; *MiBP*, mono-isobutyl phthalate; *PBDE*, polybrominated diphenyl ether; *PCB*, polychlorinated biphenyl; *PE*, pre-eclampsia; *PFAS*, per- and polyfluoroalkyl substances; PFBS, perfluorobutanesulfonic acid; *PFHxS*, perfluorohexanesulfonate; *PFOS*, perfluorooctanesulfonic acid; *PFOA*, perfluorooctanoic acid; *PFNA*, perfluorononanoic acid; *PFUA*, perfluoroundecanoic acid; *PIH*, pregnancy-induced hypertension; *P1GF*, placenta-derived growth factor; *SBP*, systolic blood pressure; *sFlt-1*, soluble fms-like tyrosine kinase 1; *TCS*, triclosan

A bold dot indicates particularly relevant references; these references are also highlighted in the reference list

**Table 2:** Pregnancy and postpartum EDC exposure and maternal weight/obesity-related outcomes

Author (year); PMID	Study sample (location)	Study design	Exposure measures and timing	Outcome measures and timing	Main findings

• Perng (2020) [54]	199 (Mexico)	Prospective cohort (ELEMENT)	Urinary BPA and phthalate metabolites in each trimester	Weight at delivery, weight change through 1 year postpartum	• Overall, EDCs associated with lower weight at delivery, but greater weight gain through 1 year postpartum (e.g., IQR increase in ΣDEHP associated with 1.38 (95% CI: 0.44, 2.33) lower weight at delivery and 1.01 (95% CI: 0.41, 1.61) kg/year slower weight loss
• Philips (2020) [53]	1213 (Netherlands)	Prospective cohort (Generation R)	Urinary BPA and phthalate metabolites at median 13 and 20 weeks gestation	Total GWG, highest GWG	• Total bisphenols and BPS associated with lower GWG, especially in normal weight women (−509 g and −398 g, respectively) • Log increase in early total bisphenol and BPA associated with lower GWG (−218, −132, respectively) in mid- to late pregnancy • Mid-pregnancy levels not associated with GWG • No associations with phthalate metabolites.
Rodriguez-Carmona (2019) [55]	178 (Mexico)	Prospective cohort (ELEMENT)	Urinary phthalate metabolites in each trimester	Weight change (per year after delivery) based on weights at 7.1 ± 1.1 years and 9.6 ± 1.5 years postpartum	• A one-unit increase in log-MCPP in pregnancy associated with 0.33 kg (95% CI: 0.09, 0.56) greater weight gain per postpartum year • A one-unit increase in log-MBzP during pregnancy associated with 0.21 kg (95% CI: −0.38, −0.03) lower weight gain per postpartum year
Bellavia (2017) [52]	347 (Boston, USA)	Prospective cohort (LIFECODES)	Urinary phthalate metabolites at median 9.9 weeks gestation	Trimester 1 BMI; early-pregnancy GWG (between 1st and 2nd prenatal visits, median 7.4 weeks)	• Higher MEP, MBzP, MCPP, and ΣDEHP associated with a rightward shift of Trimester 1 BMI • Higher MEP associated with higher GWG • Higher EDEHP associated with lower GWG
Wen (2020) [56]	613 (China)	Prospective cohort (Wuhan)	Urinary parabens in each trimester	GWG rate/week (in each trimester)	• First trimester MeP, EtP, PrP, and Σparabens associated with greater GWG rate in all trimesters (strongest in trimester 1) • Stronger associations in overweight/obese women
Shapiro (2018) [57]	1795 (Canada)	Prospective cohort (MIREC)	Urinary TCS at <14 weeks gestation	Total GWG; GWG category based on IOM recommendations	• No association between TCS and GWG
Marks (2019) [58]	905 (UK)	Prospective cohort (ALSPAC)	Serum PFAS (median 18 weeks gestation)	Absolute GWG; GWG category based on IOM recommendations	• PFOS, PFOA, and PFHxS not associated with GWG in full cohort • In under/normal weight women, higher PFNA associated with higher GWG (e.g., 10% higher PFNA associated with 0.09 kg higher GWG [95% CI: 0.02, 0.16)
Jaacks (2016) [59]	218 (Michigan and Texas, USA)	Prospective cohort (LIFE)	Pre-conception plasma PFAS	Total GWG; GWG category based on IOM recommendations; GWG AUC	• PFOS associated with AUC among women with BMI≤25 kg/m^2^ • No associations in full cohort, with other outcomes, or with other PFAS
• Ashley-Martin (2016) [60]	2001 (Canada)	Prospective cohort (MIREC)	Maternal (trimester 1) and cord blood PFAS	GWG rate (trimesters 2 and 3), total GWG; GWG category based on IOM recommendations	• Trimester 1 PFOS associated with greater GWG (*β*=0.39, 95% CI: 0.02, 0.75), particularly in underweight/normal BMI women • IQR increase (7 kg) in GWG associated with elevated cord PFOA (OR: 1.33, 95% CI: 1.13, 1.56) and PFOS (OR: 1.20, 95% CI: 1.03, 1.40)

*AUC*, area under the curve; *BMI*, body mass index; *BPA*, bisphenol A; *BPS*, bisphenol S; *DEHP*, di(2-ethylhexyl) phthalate; *EDC*, endocrine-disrupting chemicals; *EtP*, ethyl paraben; *GWG*, gestational weight gain; *IOM*, Institute of Medicine; *IQR*, interquartile range; *MBzP*, mono-benzyl phthalate; *MCPP*, mono-(3-carboxypropyl) phthalate; *MEP*, mono-ethyl phthalate; *MeP*, methyl paraben; *PFAS*, perfluoroalkyl substance; *PFHxS*, perfluorohexanesulfonate; *PFNA*, perfluorononanoic acid; *PFOS*, perfluorooctanesulfonic acid; *PFOA*, perfluorooctanoic acid; *PrP*, propyl paraben; *TCS*, triclosan

A bold dot indicates particularly relevant references; these references are also highlighted in the reference list
