# Supplementary material for: Endocrine-Disrupting Chemical Exposures in Pregnancy: a Sensitive Window for Later-Life Cardiometabolic Health in Women
Source: Curr Epidemiol Rep. Author manuscript; Available in PMC 2022 Mar 14. (PMC8920413; doi:10.1007/s40471-021-00272-7)
Supplement: Supplementary Material [file NIHMS1779070-supplement-Supplementary_Material.docx]

**Supplemental Table 1. PubMed Exposure and Outcome Search Terms**

|  | PubMed search terms |
| --- | --- |
| Endocrine Disrupting Chemicals | (endocrine disruptors[MeSH] OR phthalic acids[MeSH] OR phenols[MeSH] OR fluorocarbons[MeSH] OR flame retardants[MeSH] OR halogenated diphenyl ethers[MeSH] OR parabens[MeSH] OR endocrine disruptor*[tiab] OR persistent organic pollutant*[tiab] OR phenol*[tiab] OR bisphenol A[tiab] OR bisphenol*[tiab] OR triclosan[tiab] OR phthalic acid*[tiab] OR phthalate*[tiab] OR paraben*[tiab] OR fluorocarbon*[tiab] OR perfluorinated[tiab] OR perfluoroalkyl*[tiab] OR flame retardant*[tiab] OR polybrominated diphenyl ether*[tiab])) AND |
| Hypertensive Disorders of Pregnancy | (Pre-Eclampsia[Mesh] OR Hypertension, Pregnancy-Induced[Mesh] OR Pre eclampsia[tiab] OR preeclampsia[tiab] OR pre-eclampsia[tiab] or gestational hypertension[tiab] OR hypertensive pregnancy disorder[tiab] OR hypertensive disorders of pregnancy[tiab] OR pregnancy induced hypertension[tiab] OR pregnancy-induced hypertension[tiab]) |
| Glucose Related Outcomes | (diabetes, gestational[MeSH] OR gestational diabetes[tiab] OR ((Diabetes Mellitus[Mesh:NoExp] OR glucose tolerance test[MeSH] OR blood glucose[MeSH] OR hyperglycemia[MeSH] OR diabetes[tiab] OR diabetic[tiab] OR blood glucose[tiab] OR glucose intolerance[tiab] OR glucose tolerance[tiab] OR hyperglycemia[tiab]) AND (Pregnancy[Mesh:NoExp] OR Pregnancy Outcome[Mesh:NoExp] OR Pregnancy, High-Risk[Mesh] OR Pregnancy Complications[Mesh:NoExp] OR pregnanc*[tiab] OR pregnant[tiab] OR postpartum[tiab] OR post-partum[tiab]))) |
| Maternal Weight/Obesity Related Outcomes | ((Gestational Weight Gain[Mesh] OR gestational weight gain[tiab] OR maternal weight gain[tiab] OR GWG[tiab] OR pre-pregnancy body mass index[tiab] OR pre-pregnancy BMI[tiab] OR prepregnancy BMI[tiab] OR prepregnancy body mass index[tiab] OR ppBMI[tiab] OR postpartum weight retention[tiab] OR post-partum weight retention[tiab] OR maternal obesity[tiab])OR ((Body Mass Index[Mesh] OR Obesity[Mesh] OR Weight Gain[Mesh] OR body mass index[tiab] OR BMI[tiab] OR obesity[tiab] OR overweight[tiab] OR weight gain[tiab] OR weight retention[tiab] OR weight loss[tiab]) AND (Pregnancy[Mesh:NoExp] OR prepregnancy[tiab] OR pre-pregnancy[tiab] OR pregnancy[tiab] OR postpartum[tiab] OR post-partum[tiab] OR postnatal[tiab] OR post-natal[tiab]))) |
| Restrictions & Filters | NOT (Case Reports[ptyp] OR Comment[sb] OR Editorial[ptyp] OR Guideline[ptyp] OR Letter[ptyp] OR News[ptyp] OR Practice Guideline[ptyp] OR Review[ptyp] OR Clinical Trial[ptyp] OR “Clinical Trial, Veterinary”[ptyp] OR Randomized controlled trial[ptyp] OR “Observational Study, Veterinary”[ptyp]) NOT (Animals[Mesh] NOT Humans[Mesh]) NOT In Vitro Techniques[Mesh] AND English[lang] |
